# Visual electrophysiology in the assessment of toxicity and deficiency states affecting the visual system

**DOI:** 10.1038/s41433-021-01663-2

**Published:** 2021-07-21

**Authors:** Emily K. O’Neill, Richard Smith

**Affiliations:** 1 Clinical and Academic Department of Ophthalmology, Great Ormond Street Hospital for Children, London, UK; 2 Eye Department, Stoke Mandeville Hospital, Aylesbury, Buckinghamshire UK

**Keywords:** Eye abnormalities, Eye manifestations

## Abstract

Visual disturbance or visual failure due to toxicity of an ingested substance or a severe nutritional deficiency can present significant challenges for diagnosis and management, for instance, where an adverse reaction to a prescribed medicine is suspected. Objective assessment of visual function is important, particularly where structural changes in the retina or optic nerve have not yet occurred, as there may be a window of opportunity to mitigate or reverse visual loss. This paper reviews a number of clinical presentations where visual electrophysiological assessment has an important role in early diagnosis or management alongside clinical assessment and ocular imaging modalities. We highlight the importance of vitamin A deficiency as an easily detected marker for severe combined micronutrient deficiency.

## 摘要

由于所摄入物质的毒性或严重的营养缺乏而导致的视力损伤或障碍, 可能会给疾病的诊断和管理带来重大挑战, 例如, 怀疑患者对处方药是否有不良反应。对于视觉功能的客观评估非常重要, 尤其是在视网膜或视神经结构尚未发生改变的情况下, 因为在此阶段视觉功能的丧失可能有机会减轻或逆转。本文综述了一些临床病例, 其中视觉电生理评估在早期诊断、治疗以及临床评估中与眼部影像学一起发挥着重要作用。我们强调了维生素A缺乏症作为一种容易检测的指标做为严重复合微量营养素缺乏症标志物的重要性。

## Methodology

Searches were conducted through Medline in January 2021 using the following search strategy: Keywords: (generic drug name OR trade drug name) AND (adverse effects OR side effects OR toxic*) AND (eye diseases OR eye OR ocular OR ophthalmic). No date restriction was imposed. Results were filtered for full-text and English language and were tabulated. Papers were evaluated by relevance, study type, and quality, with articles including primary evidence prioritised. Due to the relative rarity and unpredictable incidence of many of these conditions, a large proportion of references containing primary data comprise case reports, case series, and cohorts. Review articles were prioritised by relevance to the search criteria, comprehensiveness and date of publication. References that were deemed less relevant, of lower quality or duplicated existing data were excluded.

## Introduction

Most ophthalmologists will, from time to time, encounter patients who present with persistent visual disturbance or visual failure where there is clinical suspicion either of an adverse reaction to an ingested substance or of a nutritional deficiency. Whether the onset of symptoms is acute or chronic, structural changes in the visual system such as loss of retinal photoreceptors or optic nerve fibres will often be preceded by disturbance of visual function. At a point in the clinical course where clinical examination, ocular coherence tomography, and magnetic resonance imaging as yet reveal no abnormality, there may be a window of opportunity for intervention to mitigate or reverse the effects of toxicity or rectify a nutritional deficiency before irreversible structural changes in the visual system occur. In this situation, visual electrophysiological testing may be able to provide valuable objective evidence of disordered visual function and response to treatment to supplement subjective tests of visual function such as visual acuity, perimetry, and colour vision assessment. In some instances, retinal dysfunction may occur with no, or minimal detectable structural abnormalities, making visual electrophysiology a valuable tool to enable early detection of retinal toxicity [1]. Electrophysiological testing may also be particularly important where the causal relationship between the suspected toxin or deficiency state and visual symptoms is unclear or where the patient is unable to undertake subjective tests of visual function. Rather than attempting to provide an exhaustive account of ocular toxicity and deficiency states, this paper reviews the role of visual electrophysiology testing in the diagnosis or monitoring of several important presentations which may be encountered in clinical practice.

## Chloroquine and hydroxychloroquine

Chloroquine (CQ) and hydroxychloroquine (HCQ) were introduced in 1947 and 1955, respectively for prophylaxis and treatment of malaria. Following observations that some patients taking these medications experienced improvements in arthritis and certain skin eruptions, their use expanded to conditions such as systemic and discoid lupus erythematosus and rheumatoid arthritis. Their immuno-modulatory, antimicrobial, anti-angiogenic, and anti-neoplastic properties have been studied extensively and there is considerable research interest in extending their use to the treatment of a diverse range of conditions including high-risk coronary heart disease, type 2 diabetes mellitus, primary progressive multiple sclerosis, and recurrent miscarriage [2]. CQ and HCQ are inexpensive to manufacture and are generally well tolerated. Reports of retinal toxicity in patients taking CQ appeared from the late 1950s onwards, with a typical presentation of paracentral visual field defects, progressing to central field loss and development of a “bulls-eye” maculopathy. Some patients also developed extensive peripheral field loss [3]. The pattern of retinal toxicity seen in patients taking HCQ is similar and although HCQ seems to be less toxic than CQ to the retina at doses that give an equivalent therapeutic effect, the relative risk is uncertain as accurate comparison of retrospective data is difficult [4]. The risk of retinopathy increases with dose and duration of exposure, though individual susceptibility appears to vary considerably with renal disease and concurrent use of tamoxifen identified as risk factors for toxicity [5]. Although symptomatic retinopathy in patients taking conventional doses of HCQ is uncommon, particularly when the duration of treatment is less than 5 years, Melles and Marmor found evidence of retinopathy using Humphrey 10-2 visual fields or spectral-domain ocular coherence tomography (SD-OCT) in 7.5% of 2361 patients taking HCQ for more than 5 years [5], raising the possibility that subclinical toxicity is much more common than previously thought.

The exact mechanism of CQ and HCQ toxicity in the retina remains uncertain, though photoreceptors appear to be the primary target, with the retinal pigment epithelium showing damage at a later stage [6].

**Fig. 1:**
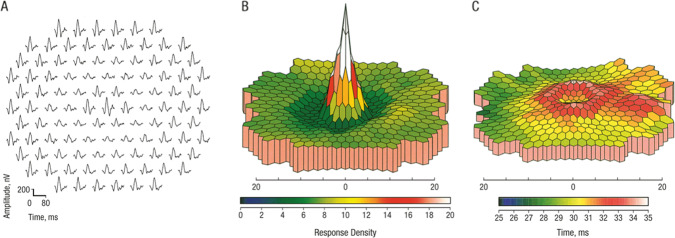
Multifocal ERG responses from one eye in a patient taking hydroxychloquine for 7.5 years who had no visible fundus abnormality. A concentric paracentral depression of the P1 component amplitude is seen in the trace array (**A**) and the three-dimensional scalar product plot (**B**). There is also mild prolongation of the P1 implicit time in the same area (**C**). From Maturi RK, Yu M, Weleber RG. Multifocal electroretinographic evaluation of long-term hydroxychloroquine users. Arch Ophthalmol. 2004;122: 973–81.

Early signs of CQ/HCQ toxicity include pericentral visual field defects with the Humphrey 10-2 protocol, perifoveal SD-OCT abnormalities (particularly disruption of the outer segment ellipsoid zone and loss of the external limiting membrane) [7]. Fundus Autofluorescence (FAF) changes (perifoveal hyper-autofluorescence with later mottled hyper- and hypo-autofluorescence) [6] and multifocal electroretinogram (mfERG) abnormalities. The mfERG is a cone-driven response recorded under light-adapted conditions from many areas of the retina simultaneously. Quantitative comparison of responses originating from the fovea and extra-foveal areas can be made by grouping individual focal responses into concentric rings and averaging the responses originating from each ring. Typically, the central response (usually designated Ring 1) corresponds to the central 3 degrees of the visual field and the next concentric ring (Ring 2) corresponds to 3-10 degrees from fixation [8]. The most reliable early sign of CQ/HCQ toxicity is a depression of the response amplitude in Ring 2 with relative preservation of the central response, expressed as the R1:R2 amplitude ratio [9] (Fig. 1).

CQ/HCQ retinopathy is irreversible and once “bulls-eye” macular changes are present, is likely to worsen for at least 3 years following cessation of the drug, though early retinopathy does not usually progress following cessation [10]. Disruption of the external limiting membrane on SD-OCT has been identified as a specific risk factor for the progression of retinopathy after cessation of treatment [6].

The Royal College of Ophthalmologists [11] has made recommendations for monitoring for CQ/HCQ retinopathy using SD-OCT and static perimetry with the 10-2 protocol as primary surveillance tools. A two-year study of patients assessed in the UK using these recommendations [12] found a prevalence of retinopathy of between 0.3% and 1.6% depending on the criteria used—considerably lower than the prevalence estimated by Melles and Marmor [5]. Although there is not currently sufficient evidence to support the use of electrophysiological testing in the routine surveillance of patients taking CQ/HCQ, where retinopathy is suspected, or where a patient has additional risk factors for developing retinopathy, mfERG can provide additional diagnostic confirmation. Cessation of CQ/HCQ therapy is not always a straightforward option, as therapeutic alternatives may be less effective or less well tolerated. In this situation, the patient may opt to continue CQ/HCQ in the presence of possible early retinopathy, in which case close surveillance including serial mfERG examinations may be required.

## Vigabatrin

Vigabatrin (VGB) is a drug used since the 1990s to treat epilepsy. In adults, it is used to manage refractory complex partial seizures which cannot be controlled through other medications, whereas in children it is a first-line option for infantile spasms associated with West syndrome. Although not proven to be effective, VGB has also been proposed as an alternative treatment for cocaine dependence [13]. It selectively and irreversibly inhibits 4-aminobutanoic acid (γ‐aminobutyric acid, GABA) transaminase, resulting in increased levels of GABA in the brain and even higher concentrations in the retina [14, 15].

First reported by Eke in 1997 [16], VGB-induced retinal toxicity has been widely documented, manifesting predominantly as bilateral irreversible peripheral visual field defects. Visual field defects vary in severity but commonly culminate in bilateral concentric constriction with temporal field and macula preservation. Patients often have normal or near-normal visual acuity (VA) and may be unaware of field loss unless it is severe [17].

Although the mechanism of toxicity is uncertain, animal studies have demonstrated VGB accumulation in many cell types of the retina and have linked retinal toxicity to photopic exposure and taurine deficiency [18]. Postmortem examination of an eye of a patient with VGB-associated visual field loss showed depletion in retinal ganglion cell and inner and outer nuclear layers [19]. There are indications from electrophysiological testing that there is dysfunction of the cone system and particularly GABAergic amacrine cells [20].

Visual field loss appears to be dose-dependent and can occur within 6 months of exposure [21]. Visual field defects may be non-progressive or may progress slowly up to 35 months, with longitudinal studies showing that the loss typically persists indefinitely following cessation of VGB therapy [21]. A few isolated cases of improvement following cessation in children have been reported but may be artefactual as a result of learning effects. These findings have led to recommendations to limit dosage to 3 g/day in adults and 50–100 mg/kg/day in children as a maintenance dose, with withdrawal encouraged if there is no clinical benefit [22].

Estimates of the prevalence of VGB-induced retinal toxicity vary, but a systematic review by Maguire estimated a prevalence of 52% [95% confidence interval (CI) 46–59] for adults and 34% (95% CI 25–42) for children [23]. Prevalence increases with age and cumulative dose and may be higher in males [21].

Identifying early signs of VGB-induced retinal toxicity using visual field assessment remains a challenge as perimetry is often not suitable for children and patients with cognitive impairment, learning difficulties, or fatigue, all of which are frequently reported in epilepsy [24]. Furthermore, perimetry alone may not detect subtle peripheral field changes and apparent field constriction is not necessarily indicative of toxicity. The Royal College of Ophthalmologists has recommended that all patients taking VGB undergo a complete eye examination including visual field assessment (preferably gradient adapted static perimetry) before starting treatment, with perimetry repeated every 6 months for 5 years [22]. An additional threshold test extending to 30 degrees of eccentricity should be conducted if a field defect is reported [22].

Due to the limitations of visual field assessment in paediatric patients and approximately 20% of adult epilepsy patients [25], additional tests may be utilised to detect toxicity, including fundoscopy, optical coherence tomography (OCT), and electrophysiology. Fundoscopy is often unremarkable in patients with VGB-associated visual field loss, although macular pigment epithelial changes and thinning of the nasal retinal nerve fibre layer (RNFL), termed ‘reverse optic atrophy’ have been reported [15]. Often these changes are subtle or only evident where there is severe field loss. OCT allows quantitative evaluation of the RNFL, and reduced RNFL thickness has been shown to correlate with visual field defects [25]. Despite the cooperation requirements of OCT, handheld devices can yield reliable measurements in children taking VGB [26]. Alternative VEP methods of visual field assessment for children taking VGB have been explored [27] but have yet to be validated for routine clinical practice.

As VGB toxicity appears to be predominantly an inner retinal dysfunction, the standard full-field electroretinogram (ERG) typically shows only subtle abnormalities which may not correlate with the severity of visual field loss [28]. However, reduced amplitude of the 30 Hz flicker ERG (a cone system-driven response, originating in the inner retina) and the photopic negative response (PhNR) (which reflects ganglion cell activity) have been reported and may provide useful supporting evidence of toxicity in infants and children [29, 30]. Although the ERG does not usually provide unequivocal evidence of VGB toxicity, it is valuable in the initial assessment of a patient receiving VGB to exclude other retinal causes of visual field constriction such as photoreceptor dystrophies. Electrophysiological assessment may also help to identify pre-existing ERG abnormalities, possibly associated with other anti-epilepsy medications, which were found in 30% of patients prior to VGB treatment by Westall et al. [29]. There is, however, currently insufficient evidence to recommend electrophysiological assessment as part of the routine ongoing monitoring of visually asymptomatic patients taking vigabatrin.

## Ethambutol

Ethambutol was introduced in the early 1960s for the treatment of tuberculosis and other mycobacterial infections. The first report of optic neuropathy in patients treated with ethambutol appeared whilst the drug was still in clinical trials, with optic disc hyperaemia and swelling and the appearance of a centrocaecal scotoma, which resolved following cessation of treatment [31]. The incidence of optic neuropathy appears to be dose and duration-dependent, with a reported incidence of 45% at doses of 60–100 mg/kg/day [31] reducing to less than 1% with a dose of 15 mg/kg/day [32]. However, there does not appear to be a “safe” dose, and optic neuropathy has been reported with a dose of only 3.6 mg/kg/day after 6 months of treatment [33]. The toxicity of ethambutol may be potentiated by renal impairment or co-administration of other drugs which can cause optic neuropathy in their own right, such as linezolid [34] or isoniazid [35].

In a series of 857 patients taking ethambutol, the incidence of optic neuropathy was 1.5%, and of these, reduced visual acuity was evident in 65% and abnormal colour vision in 61% [36]. Colour vision abnormalities ranged from mild red-green defects to complete loss of colour vision.

Visual field defects attributed to ethambutol toxicity are typically central or centrocaecal, but can also be extensive with homonymous or bitemporal patterns, raising the possibility that the optic chiasm or optic tracts may be affected in some cases [36–39]. Peripapillary RNFL thinning, particularly in the temporal quadrant and macular nerve fibre layer thinning may be evident on OCT [33, 40].

Where the visual acuity is substantially reduced due to ethambutol optic neuropathy, the pattern visual evoked potential (PVEP) may be reduced in amplitude or unrecordable. Where a PVEP is recordable, a delay in the P100 component is the most consistent abnormal finding [33, 38, 41]. Multifocal ERG abnormalities [42] and full-field ERG abnormalities (reduced scotopic and photopic b-wave amplitudes and loss of oscillatory potentials) [33] have also been reported, which suggests that the toxic effect of ethambutol in the retina may not be confined to the ganglion cells but may involve other cell types such as amacrine and bipolar cells.

Ethambutol appears to render ganglion cells abnormally sensitive to glutamate ions (an excitotoxic effect), leading to a rise in intracellular calcium ions which disrupts mitochondrial function [43]. This raises the possibility that drugs that inhibit the NMDA subtype of glutamate receptor, such as memantine and amantadine may protect against ethambutol toxicity and there is evidence that this may be the case in animal models [43, 44].

Although the prognosis for recovery from ethambutol optic neuropathy is generally good following cessation of the drug, permanent visual loss can occur even when ethambutol is discontinued promptly [31]. Kim et al found significant delays in PVEP P100 latency at 2 months and 4 months of therapy with ethambutol even where visual acuity and colour vision were normal, suggesting that subclinical toxicity is common [45]. Serial monitoring of asymptomatic patients taking ethambutol with PVEP and OCT retinal nerve fibre layer thickness is a practical addition to the recording of visual acuity and colour vision during treatment and should be considered, particularly for patients who may require prolonged treatment with ethambutol. Where symptomatic ethambutol toxicity is suspected, electrophysiological assessment may also include PERG or mfERG and full-field ERG.

## Quinine

Quinine, occurring naturally in the bark of the cinchona tree, has been used from antiquity to treat malaria. It is still sometimes used for the treatment of malaria caused by *Plasmodium falciparum* and, at a lower dose, for the treatment of nocturnal leg cramps, though its use for both of these indications is declining where safer alternatives are available. It is also used at a lower concentration in tonic water to impart a bitter flavour. It has a narrow therapeutic index and accidental or intentional overdosage with quinine may result in hypotension, life-threatening cardiac arrhythmias, and seizures [46]. Acute overdosage (which may be accidental, or a result of attempted suicide) can also result in severe visual loss, sometimes to no perception of light bilaterally within a few hours of ingestion. In the acute presentation, the pupils are typically fixed and semi dilated. The retina may appear normal initially, though there may be transient retinal pallor with the appearance of a cherry-red spot at the fovea [47, 48].

Later findings typically include arteriolar attenuation, optic disc pallor, and pronounced thinning of the inner retinal layers on SD-OCT [47, 49]. There may be spontaneous visual improvement in the days or weeks after the overdose, sometimes to a normal visual acuity, but with night-blindness and pronounced concentric constriction of visual fields which does not improve [48].

The mechanism of damage to the retina in quinine poisoning probably involves direct toxicity to the Müller cells, bipolar cells, amacrine cells, and ganglion cells [50], though the photoreceptors may also show evidence of damage on SD-OCT [47]. The reason why central retinal function may improve when peripheral retinal function does not is unknown. No effective antidote to the retinal toxicity of quinine has yet been found.

In the first few days following ingestion of quinine, the bright flash dark-adapted ERG may show a delayed, supernormal a-wave and b-wave with absent oscillatory potentials [51]. Later, the bright flash dark-adapted ERG shows a reduced b-wave with a relatively preserved a-wave, producing an “electronegative” waveform [49, 51, 52]. The single-flash photopic ERG shows a prolonged a-wave trough and small, steeply rising b-wave. The 30 Hz flicker photopic ERG is reduced in amplitude and delayed, and the long (200 ms) flash photopic ERG has a characteristic abnormal shape with a positive plateau following the off d-wave peak, indicative of cone ON- and OFF-pathway dysfunction [49, 50]. The pattern ERG (reflecting macular function) may be absent even in the presence of good visual acuity [49]. The ERG changes persist indefinitely, which can help distinguish the condition from retinitis pigmentosa where a history of quinine ingestion may not be volunteered by the patient [53].

## Iron chelators

Iron-chelating agents are used to prevent iron accumulation in thalassaemia and other conditions which require frequent blood transfusion. Many tissues and organs are vulnerable to iron overload, especially the heart, liver, bone and endocrine tissues. The most commonly used and studied iron chelator is desferrioxamine mesylate (DFO); however, as it can only be administered parenterally, its use has declined with the introduction of newer oral chelators deferiprone and deferasirox.

Since the 1980s, single case reports and small case series have reported ocular toxicity associated with DFO, with reported findings of pigmentary retinopathy or optic neuropathy accompanied by reduced visual acuity and colour vision, nyctalopia, cataract, and visual field defects [54, 55]. The primary target of DFO toxicity appears to be the retinal pigment epithelium (RPE). The mechanism is poorly understood but may involve both direct chelation of iron stores within the RPE and secondary depletion of trace elements. Early signs of toxicity include transient opacification of the RPE and outer retina, followed later by pigmentary change [54]. The fundoscopic appearance of DFO retinopathy is variable, typically resembling a pattern dystrophy, however vitelliform and “bulls-eye” lesions have also been described [56, 57]. Pigmentary changes in the macula may be subtle and easily missed on fundoscopy. Historically, fluorescein angiography (FA) was widely utilised to diagnose DFO retinopathy [54], but FAF, confocal scanning laser ophthalmoscopy, and SD-OCT are now more widely used in the detection of subtle retinal abnormalities and to monitor progression over time [57, 58].

Reported electrophysiological abnormalities in acute DFO toxicity include markedly reduced scotopic ERG a- and b-wave amplitudes, delayed photopic cone b-waves, and a reduced electrooculogram (EOG) light peak to dark trough ratio [54, 59]. Dettoraki [60] found that the full-field ERG and mfERG were more sensitive in detecting early toxicity than VEP, FAF, and OCT. Where there are visible pigmentary changes involving the macula, the mfERG typically shows reduced responses in the central rings, though such changes are not unique to DFO toxicity [57].

Some reports describe reversibility of visual defects when DFO therapy is changed from intravenous to subcutaneous administration [61] or is completely stopped [62], but permanent visual deterioration or progression of retinopathy after DFO has been discontinued has also been documented [59]. The paucity of longitudinal studies means that there is limited information on the incidence of ocular toxicity associated with iron chelators, although it appears to be a rare finding. A 22-year review of 88 patients receiving chelation therapy reported only 3 patients with suspected toxicity [63].

The lack of a formal definition of DFO ocular toxicity and evidence-based guidelines for screening means that arrangements for surveillance of patients taking DFO are typically determined by local protocols between prescribers and ophthalmology services. To minimise the risk of toxicity, it has been recommended that dosage should not exceed 50 mg/kg of body weight in adults and 25–30 mg/kg in children [64], and regular examination using fundoscopy, FAF, and OCT is encouraged to identify early signs of retinal and RPE damage.

It is unclear if the long-term use of newer iron-chelating agents deferiprone and deferasirox will result in similar retinal toxicity. There are a few case reports of macular changes in patients taking Deferasirox, with evidence suggesting that vision can improve after therapy is ceased [65]. Further data is required on the ocular safety of oral iron chelators and ophthalmology services are likely to receive requests to monitor patients receiving these agents. Clinical decisions to continue, change, or discontinue therapy may be difficult because of the need to balance the risks of irreversible organ damage from iron overload with a risk of retinal toxicity. Based on the reported electrophysiological abnormalities in patients taking DFO, the most useful electrophysiological tests for monitoring patients receiving iron chelation therapy are likely to be the full-field ERG, with the addition of either the PERG or mfERG to assess macular toxicity.

## Ocular siderosis

Iron is a component of haemoglobin, and its capacity to mediate electron transfer makes it a component or co-factor in many enzymatic processes, including those involved in oxidative phosphorylation in mitochondria and RPE65. The ferrous (Fe^2+^) ion is an electron donor and readily catalyses the conversion of hydrogen peroxide into the hydroxyl radical (OH^·^) which is highly reactive and causes oxidative damage to proteins, DNA, and lipids. Almost all the body’s iron is normally bound to protein, but the mechanisms for quenching free radicals and removing iron from the eye may be overwhelmed by the presence of a retained ferrous intraocular foreign body.

Iron-containing foreign bodies are particularly liable to enter the eye during activities that involve striking metal on metal, such as using a hammer and cold-chisel [66]. A shard of metal generated in this way may have sharp edges and sufficient momentum to penetrate the cornea or sclera.

Larger foreign bodies tend to cause symptomatic intraocular haemorrhage or rapidly evolving cataract, but smaller fragments may lodge in the ciliary body, iris, lens, or anterior chamber drainage angle with few, if any, short-term symptoms. If the injury has gone unnoticed by the patient or if clinical assessment has failed to identify an entry wound, the undetected ferrous foreign body will release iron as it slowly degrades.

The features of established ocular siderosis include cataract with brown deposits on the lens near the pupil margin, brown or greenish discolouration of the iris, a dilated tonic pupil with light-near dissociation, brown discolouration of the corneal stroma, and increased pigmentation of the trabecular meshwork, diffuse retinal pigmentary changes, retinal arteriolar attenuation and cystoid macular oedema. Not all of these features will necessarily be present, and the changes can be subtle. Signs of siderosis can become evident anywhere from a few weeks to several years after the injury [67].

The bright flash dark-adapted ERG b-wave amplitude has been reported to be transiently supernormal (125% or more of that of the fellow eye) with a normal a-wave in some cases of early siderosis [66]. The reason for this is uncertain, but as the condition advances, the rod and cone b-waves, the oscillatory potentials, and the 30 Hz flicker response become progressively attenuated with relative preservation of the a-wave, suggesting that the inner retina is affected earlier than the photoreceptors. Ultimately the scotopic and photopic ERG may become undetectable. The time between the earliest detectable ERG changes and extinction of the ERG varies but can be less than 18 months [68, 69]. Following surgical removal of the foreign body, ERG changes did not recover in 6 cases reported by Hope-Ross [69] even though the final visual acuity was 6/12 in all cases.

There is evidence that delayed removal of an intraocular foreign body may be followed by continuing deterioration of the ERG [70]. However, substantial recovery of the ERG was reported following surgery at about a month post-injury by Imaizumi et al. [71] in a young adult, although the oscillatory potentials and 30 Hz flicker amplitudes did not normalise. These cases suggest that identification and early removal of an iron-containing intraocular foreign body before clinical or electroretinographic signs of siderosis appear may be important for the prevention of ocular siderosis. However, this goal is not always achievable as patients may decline surgery or the foreign body may be difficult to locate or inaccessible. Small foreign bodies buried in the pars plicata of the ciliary body can be particularly difficult to locate, even with the assistance of modern imaging equipment. Hwang records a case of a ferrous foreign body embedded in the anterior part of the optic nerve with good visual acuity. A decision was made to leave the foreign body in situ and there was no evidence of siderosis after 2 years [72]. In a situation where surgery to remove a foreign body carries a high short-term risk to vision, it may be reasonable to monitor the patient with serial ERGs and re-evaluate the risks and benefits of surgery should ERG changes suggestive of early siderosis appear.

Electroretinography may also provide useful prognostic information in delayed presentations of established siderosis, including patients who present with a dense cataract and an uncertain history of previous trauma. Mild or moderate ERG abnormalities may be compatible with good vision [69] though an extinguished ERG suggests a poor prognosis for recovery of vision.

## Methanol

Methanol is found in some brands of antifreeze and is used as an ingredient of methylated spirits to denature ethanol, rendering it unfit for human consumption, whilst still allowing it to be used as a solvent or a fuel. Methanol may also be a contaminant in illegally distilled spirits, which has resulted in mass-poisonings [73]. The effects of accidental or intentional ingestion of methanol are dose-related and include blindness, acute encephalopathy (which may be fatal), and metabolic acidosis. Methanol is less intoxicating than ethanol, so victims may report early symptoms at a point when intervention may be effective.

Methanol is oxidised to methanal in the liver by alcohol dehydrogenase and undergoes further enzymatic oxidation to methanoic (formic) acid, which inhibits cytochrome-c oxidase, thereby reducing ATP production in mitochondria in metabolically active sites such as ganglion cell axons. Hayreh et al. demonstrated in rhesus monkeys that, following ingestion of methanol, a combination of arrest of axonal transport and swelling of oligodendrocytes in the retrolaminar optic nerve produces a clinical picture resembling severe papilloedema which develops gradually over 1–2 days [74].

Accounts of acute methanol poisoning in humans show similar findings and have a similar time course, with gradual resolution of optic disc swelling and development of optic atrophy over 1–2 months [75]. The optic discs may later develop pronounced cupping, resembling advanced normal-tension glaucoma, which suggests that there is also loss of glia in the prelaminar optic nerve [76, 77].

Presenting visual symptoms range from blurring or greying of vision to complete loss of vision, and a central scotoma may be evident, but visual improvement has been documented as disc swelling subsides [75]. The severity of metabolic acidosis at presentation appears to correlate with the severity of visual loss and electrophysiological abnormalities [74, 78].

Urban et al. found pattern-reversal visual evoked potentials (PVEP) abnormalities in 43% of a cohort of 47 victims of a mass-poisoning event, with a mild or moderate delay of the P100 component in subjects (typically 118–130 ms) in subjects where the P100 amplitude was preserved, though the PVEP was unrecordable in those with severe visual loss, as expected [73]. Five subjects had a delayed PVEP despite normal visual acuities. There was a correlation between the biochemical severity of poisoning and VEP abnormalities.

Reported full-field ERG abnormalities following methanol poisoning include selective reduction of the b-wave [79], combined scotopic a-wave and b-wave reduction and reduced 30 Hz flicker amplitude [80] which suggests that methanol is also toxic to the retina.

The treatment of methanol poisoning includes resuscitation, correction of metabolic acidosis, and administration of fomepizole (4-methylpyrazole), an inhibitor of alcohol dehydrogenase, which delays the conversion of methanol to its more toxic metabolites. Electrophysiological assessment of patients with known or suspected methanol poisoning should include PVEP (and flash VEP where the PVEP is unrecordable), PERG and full-field ERG.

## Toxic-nutritional optic neuropathy

This term is used to describe a bilateral optic neuropathy of gradual onset in adults characterised by reduced contrast sensitivity, dyschromatopsia, and the appearance of central or centrocaecal scotomas. Descriptions of this clinical presentation appeared from the mid-18th Century onwards, but the first systematic description is attributed to de Schweinitz in 1896 [81] and anecdotal observations that heavy alcohol or tobacco consumption seemed to be associated with the condition led to the older term ‘tobacco-alcohol amblyopia’. Several epidemics with a similar pattern of optic neuropathy have occurred, notably in Cuba in 1992–93 when more than 50,000 cases of optic neuropathy associated with peripheral and auditory neuropathy were linked to deficiencies in B vitamins and folate during an economic embargo [82], raising the possibility that the condition is caused primarily by dietary deficiency of B vitamins or other micronutrients, given that these are common in people who are heavily dependent on alcohol.

Although the onset of toxic-nutritional optic neuropathy is usually insidious with few fundoscopic signs, transient optic disc swelling and telangiectases, and peripapillary haemorrhages sometimes occur [83], prompting comparisons with methanol and ethambutol optic neuropathy and Leber hereditary optic neuropathy (LHON).

The most likely mechanism which links possible toxic, nutritional, and genetic aetiologies of this clinical entity is decompensation of aerobic respiration in the mitochondria of ganglion cells which serve the central visual field. Carbon monoxide, cyanide ions (both components of cigarette smoke), and methanoic acid (a metabolite of methanol) exert a direct effect on the cytochrome enzymes. The antibiotic linezolid probably interferes with oxidative phosphorylation indirectly via inhibition of mitochondrial ribosomes [35]. Ethambutol probably exerts an indirect excitotoxic effect on oxidative phosphorylation by increasing intracellular calcium via the NMDA glutamate receptor [43]. Thiamine, riboflavin, niacin, pyridoxine, vitamin B12, folate, and copper are all required for mitochondrial metabolism [84]. The G3460A, G11778A and T14484C mutations of mitochondrial DNA responsible for >90% of cases of LHON all disrupt the function of Complex 1 of the electron transport chain [85]. The OPA1 gene encodes a GTPase which is involved in the control of mitochondrial fusion and fission and mutations in this gene have been documented to cause autosomal dominant optic atrophy [86].

In addition to measurement of visual acuity, colour vision, and visual fields, imaging and visual electrophysiology can be helpful. SD-OCT may show RNFL thinning which is not necessarily limited to the temporal quadrant [87].

The a-wave and b-wave of the dark adapted and light-adapted full-field ERG are likely to be normal, but the amplitude of the photopic negative response (PhNR) of the full-field ERG which reflects ganglion cell function is likely to be reduced, as is also the case in LHON [88]. The pattern ERG (PERG) is a macular-dominated response and usually shows preservation of the P50 component with selective reduction of the N95 component, reflecting abnormal ganglion cell function [89]. The PVEP to a reversing checkerboard stimulus typically shows predominantly a reduction in amplitude of the P100 component, although delays in latency may also be seen [89]. When the visual acuity deteriorates to 6/60 or worse, the PVEP even to large (60’) checks may be unrecordable.

An individual presenting with toxic-nutritional optic neuropathy may have more than one risk factor for ganglion cell mitochondrial decompensation, for example, a diet deficient in B-complex vitamins and heavy tobacco consumption. These can potentially be addressed to prevent further deterioration of vision. However, there may also be underlying genetic factors that influence the level of individual vulnerability to optic neuropathy.

Electrophysiological assessment of patient with suspected toxic-nutritional optic neuropathy should include PVEP (and flash VEP where the PVEP is unrecordable), PERG and full-field ERG, ideally including measurement of the photopic negative response.

## Micronutrient deficiencies

Many micronutrients are essential to maintain the functioning of the visual system and prolonged deficiencies may cause vision loss and affect the structural integrity of the eye. The prevalence of micronutrient deficiencies may be increasing, due to the rise in malabsorption syndromes secondary to inflammatory bowel diseases and gastric bariatric surgery, the popularity of strict vegan and vegetarian diets, high rates of alcohol dependency, and avoidant restrictive food intake disorder (ARFID).

Although ARFID is a relatively new diagnosis, emerging evidence suggests that ARFID in Autistic Spectrum Disorder (ASD) is a significant and rising cause of micronutrient deficiency in paediatric populations [90, 91]. Patients with ARFID may avoid particular foods due to associations with a specific sensory aspect such as colour or texture, fear of contamination, or past traumatic events related to food [92]. Individuals with restricted diets often have multiple vitamin and trace element deficiencies, including vitamin A, B9, B12, C, D, E, zinc, iron, and copper, of which the mechanisms of interaction are not fully understood.

Although there are no population studies of ARFID, Hamilton et al. [93] reported 3 cases of vision loss secondary to highly restrictive dietary intake in patients with ASD. The causes of reduced vision were retinal dysfunction, optic neuropathy, dysfunction, or a combination of these, and visual loss was severe in 2 cases. All patients were deficient in vitamin A. A review of a larger series of cases is in progress and vitamin A deficiency was prominent feature of most of these (Ruth Hamilton, personal communication).

Vitamin A deficiency (VAD) retinopathy is the most extensively reported nutritional retinopathy. Vitamin A is essential for rhodopsin and cone opsin formation and the maintenance of corneal and conjunctival epithelial cell function. Patients with VAD can present with nyctalopia, xerophthalmia, ocular surface keratinisation (Bitot spots), and retinal white spots, although ocular examination may show no abnormality [94, 95].

**Fig. 2:**
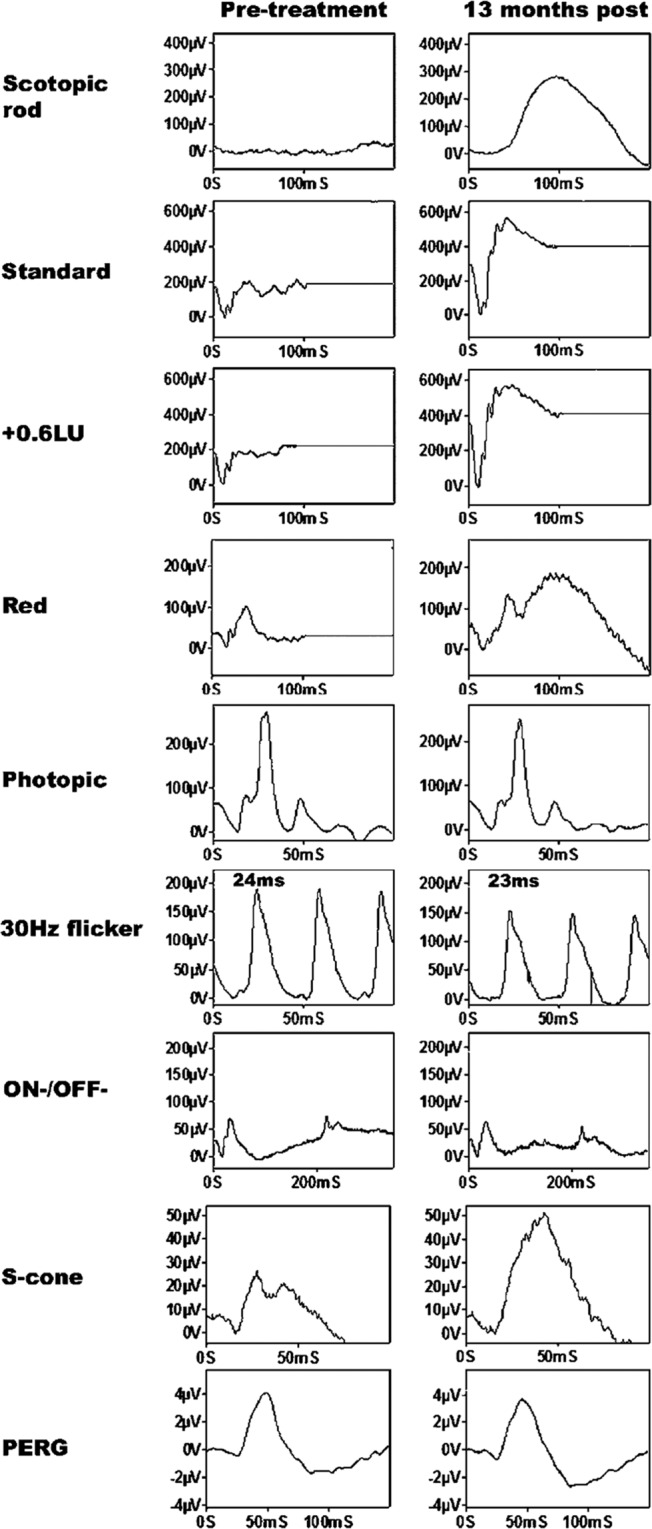
Full-field ERG responses from a patient with Vitamin A deficiency before, and after replacement treatment showing full recovery of the dark adapted ERG. The use of a red flash under dark adapted conditions (‘Red’) demonstrates that the residual dark adapted ERG response prior to Vitamin A replacement originates wholly from the cone system. From McBain, V., Egan, C., Pieris, S. et al. Functional observations in vitamin A deficiency: diagnosis and time course of recovery. Eye. 2007;21:367–76.

The ERG in VAD shows characteristic abnormalities. The response to a rod-specific stimulus is absent or markedly reduced and the bright-flash dark- adapted ERG shows a contribution from dark-adapted cones, with loss of the rod system component. The light-adapted transient and 30 Hz flicker ERG are relatively preserved but reduced in amplitude [96]. The ERG to a red flash under dark-adapted conditions is particularly useful to identify selective rod dysfunction in these patients and can be used to verify the cone system origin of residual scotopic bright flash ERG response [97] (Fig. 2). The dark-adapted ERG responses typically show recovery following prolonged dark adaptation. Dark adaptometry confirms delayed recovery from bleach, particularly, though not exclusively for the rod component of the dark adaptation curve [98]. Additional changes in s-cone function, mfERG, microperimetry, and pERG have been described in VAD patients [95, 96].

Identifying characteristic rod dysfunction through visual electrophysiology enables early detection and treatment for VAD retinopathy and associated micronutrient deficiencies, which is important as vision loss is often reversible through early and adequate supplementation. ERG and dark adaptometry studies have demonstrated the recovery of rod function and are valuable as a monitoring tool once treatment is initiated [95, 98].

The full impact of nutritional deficiencies on the visual system is still unknown. Severe combined micronutrient deficiency as reported in children with ARFID can have a major and lasting impact on visual function. The high prevalence of VAD in these children suggests that behavioural or electroretinographic evidence of vitamin A deficiency should be regarded as a ‘red flag’ for specialist referral, even if the vision is otherwise good.

Electrophysiological assessment of patients with suspected VAD or severe combined micronutrient deficiency, even where there is no clinical evidence of impaired night vision, should include a full-field ERG, including dark-adapted responses to a red flash and, where possible, a full-field ERG following prolonged dark adaptation. Where visual acuity is reduced, the assessment should also include PERG, PVEP (and, where the PVEP is unrecordable, a flash VEP).

## Summary

The referral of a patient with visual loss associated with possible exposure to a toxic substance or a severe deficiency can be challenging for clinicians, especially when symptoms are acute, associated with a prescribed medicine, or are related to self-harm. If symptoms have arisen more gradually, the nature and extent of exposure to a suspected toxin may be difficult to ascertain. Where the suspected toxin is a prescribed medicine, a decision to reduce the dose or discontinue therapy may not be straightforward because it may precipitate a relapse of the condition for which it was prescribed, and alternative therapies may be less well tolerated.

Under these circumstances, it is desirable to establish a link between the suspected toxin or deficiency state and the visual symptoms with as high a degree of certainty as possible. However, this process may be complicated by interactions between potential toxins, deficiency states, and individual genetic variation, or by a lack of detailed information about the circumstances of exposure (for instance, where illicit substances may have been involved).

Visual electrophysiology testing can play an important role in providing evidence to establish a causal connection, particularly where structural changes have not yet evolved, where subjective tests of visual function are unreliable, or when investigating the effects of prenatal exposure to potential toxins on postnatal visual development. It may also have an important role in monitoring a response to intervention or supporting clinical decision-making where withdrawal of a prescribed medication is undesirable or has been declined by the patient. Selection of an appropriate range of electrophysiological tests allows the function of the visual pathway from the photoreceptors and RPE to the primary visual cortex to be probed, and regional disturbances of retinal function to be elicited (Table 1).

**Table 1:** Summary of main target tissues of ocular toxicity and deficiency states, key investigations and the role of visual electrophysiological assessment.

Toxin/deficiency state	Target	Key investigations	Role of electrophysiology
Chloroquine and Hydroxychloroquine	PR and RPE	Perimetry, SD-OCT, FAF, mfERG	Confirmatory, monitoring
Vigabatrin	RGC, amacrine cells	Perimetry, SD-OCT, ERG including 30 Hz flicker and PhNR	Exclusion of other retinopathies, confirmatory (second line)
Ethambutol	RGC, possibly amacrine and bipolar cells	Perimetry, colour vision tests, SD-OCT, VEP	Confirmatory, monitoring
Quinine	Muller cells, RGC, amacrine cells, bipolar cells	Perimetry, SD-OCT, ERG including 30 Hz flicker and long flash, PERG	Diagnosis, monitoring
Iron Chelators	RPE, PR	Perimetry, colour vision tests, FAF, SD-OCT, ERG, mfERG,PERG	Confirmatory, monitoring
Ocular Siderosis	All retinal layers, RPE	ERG including oscillatory potentials	Diagnosis, monitoring
Methanol	RGC, probably other retinal cells	Perimetry, VEP, PERG, ERG including PhNR	Confirmatory, monitoring
Toxic-nutritional Optic Neuropathy	RGC	Contrast sensitivity, colour vision tests, SD-OCT, ERG including PhNR, PERG, VEP	Confirmatory
Vitamin A deficiency	Conjunctiva, cornea, PR (especially rods)	ERG including dark adapted red flash, dark adaptometry	Diagnosis, monitoring
Severe combined micronutrient deficiency (eg ARFID)	PR, RGC	Colour vision tests, ERG, VEP	Diagnosis, monitoring

The role of visual electrophysiology is “diagnostic” where the changes are specific for the condition in question, and “confirmatory” where the changes are not specific to the condition in question but are likely to support or exclude the diagnosis in combination with other investigations.

*ARFID* avoidant restrictive food intake disorder, *RPE* retinal pigment epithelium, *PR* photorececeptors, *RGC* retinal ganglion cells, *ERG* full-field electroretinogram, *mfERG* multifocal electroretinogram, *EOG* electrooculogram, *VEP* visual evoked potential, *PhNR* photopic negative response, *PERG* pattern electroretinogram, *SD-OCT* spectral-domain ocular coherence tomography, *FAF* fundus autofluorescence.

This paper has reviewed a number of clinically important toxicity or deficiency states involving the visual system where there is evidence that visual electrophysiological testing can assist the clinician. New hazards and side-effects of new treatments which affect the visual system are likely to emerge in the future and visual electrophysiology will continue to play a part in establishing causal connections with presenting symptoms, identifying the mechanism of damage to the visual system, and monitoring the response to intervention.
